# 4D-Printed Magnetic Responsive Bilayer Hydrogel

**DOI:** 10.3390/nano15020134

**Published:** 2025-01-17

**Authors:** Yangyang Li, Yuanyi Li, Jiawei Cao, Peng Luo, Jianpeng Liu, Lina Ma, Guo-Lin Gao, Zaixing Jiang

**Affiliations:** 1 MIIT Key Laboratory of Critical Materials Technology for New Energy Conversion and Storage, School of Chemistry and Chemical Engineering, Harbin Institute of Technology, Harbin 150001, China; liyangyanghit2024@163.com (Y.L.); 18577910619@163.com (Y.L.); fishcho@126.com (J.C.); 2 Jianghuai Advance Technology Center, Hefei 230009, China; luop@tju.edu.cn (P.L.); 15549416750@163.com (J.L.); 3 College of Chemistry and Chemical Engineering, Qingdao University, Qingdao 266071, China; malina@qdu.edu.cn

**Keywords:** intelligent hydrogel, magnetic responsive gels, 4D printing

## Abstract

Despite its widespread application in targeted drug delivery, soft robotics, and smart screens, magnetic hydrogel still faces challenges from lagging mechanical performance to sluggish response times. In this paper, a methodology of in situ generation of magnetic hydrogel based on 3D printing of poly-N-isopropylacrylamide (PNIPAM) is presented. A temperature-responsive PNIPAM hydrogel was prepared by 3D printing, and Fe_2_O_3_ magnetic particles were generated in situ within the PNIPAM network to generate the magnetic hydrogel. By forming uniformly distributed magnetic particles in situ within the polymer network, 3D printing of customized magnetic hydrogel materials was successfully achieved. The bilayer hydrogel structure was designed according to the different swelling ratios of temperature-sensitive hydrogel and magnetic hydrogel. Combined with the excellent mechanical properties of PNIPAM and printable magnetic hydrogel, 4D-printed remote magnetic field triggered shape morphing of bilayers of five-petal flower-shaped hydrogels was presented, and the deformation process was finished within 300 s.

## 1. Introduction

With the rapid development of science and technology, intelligent materials have become an important direction of material science research [1]. These materials can sense and respond to environmental changes and can self-regulate according to external stimuli, thus showing great application potential in various fields [2,3,4,5,6,7]. 4D printing, as an emerging technology, gives 3D-printed objects the ability to automatically deform under specific conditions by taking time as the fourth dimension, making the application prospect of smart materials broader [8,9,10,11]. Among many smart materials, wettable materials show significant advantages in breaking through the single wettability limitations of traditional materials. Its surface-wetting properties can be regulated in the range of super hydrophobic to super hydrophilic, so it has a wide application prospect in many fields [12,13]. For example, in terms of oil and water separation, intelligent wetting materials can effectively distinguish and separate oil and water, improving the separation efficiency [14,15]. In addition, such materials also play an important role in cell adhesion and release, and by regulating surface infiltration, the adhesion and detachment of cells on the surface of the material can be controlled, thus providing a new means in cell culture and tissue engineering [16,17]. In the field of biological detection, intelligent infiltrating materials can enhance the sensitivity and accuracy of detection through changes in surface wettability [18].

Temperature-sensitive gel is a kind of smart material that can react to a temperature-changing field. It is unique in that it combines temperature-sensitive properties and can exhibit different physical and chemical properties under different temperature conditions [19,20]. The basis of this material is a temperature-sensitive polymer, which can be controlled in response to external stimuli through fine molecular design and microstructural regulation. Thermosensitive polymers can change at specific temperatures, which makes them great application potential in biomedicine, flexible electronic devices, environmental monitoring, and other fields.

4D-printed magnetic hydrogels have shown potential application prospects in targeted drug delivery [21], soft robotics [22], smart screens [23], and others [24]. Magnetic hydrogels can be used to create smart drug delivery systems that can control the release rate and location of drugs based on stimulation from external magnetic fields or physiological signals such as pH or temperature changes. In tissue engineering, hydrogels act as scaffold materials to support the growth and differentiation of cells and promote the repair and regeneration of damaged tissues. Through 4D printing technology, tissue engineering scaffolds with complex structures and specific functions can be manufactured. In addition, when magnetic hydrogels function as manufactured environmental monitoring sensors, sensor arrays with high sensitivity and selectivity can be manufactured through 4D printing technology. However, 4D printing magnetic hydrogels also faces some limitations and challenges, such as limited material choices, difficulty in controlling printing accuracy, and strong environmental dependence.

This paper focuses on the application of magnetic hydrogels in shape deformation. Magnetic Fe_2_O_3_ particles were uniformly dispersed throughout a poly-N-isopropylacrylamide hydrogel (Fe_2_O_3_ + PNIPAM) via in situ precipitation. By employing bilayer hydrogel 4D printing technology, we leveraged the difference in shrinkage rates between the two layers to achieve directional deformation. This was done by combining temperature-sensitive and magnetic hydrogels, creating a smart response system suitable for extrusion 3D printing manufacturing. The resultant bilayer hydrogel could change its shape in response to external magnetic stimuli. This work offers new insights for the future design and development of smart hydrogels.

## 2. Materials and Methods

### 2.1. Materials

Poly (N-isopropyl acrylamide) (PNIPAM, 99%, Macklin, Shanghai, China), dimethyl aminopropyl methacrylamide (DMAPMA, 97%, Sigma-Aldrich, St. Louis, MO, USA), Laponite XLG (XLG, 99%, Macklin, Shanghai, China), N,N’-Methylenebisacrylamide (BIS, 99%, Macklin, Shanghai, China), diphenyl(2,4,6-trimethylbenzoyl) phosphineoxide (TPO, 97%, Macklin, Shanghai, China), Iron(II) chloride tetrahydrate (FeCl_2_·4H_2_O, 98%, Macklin, Shanghai, China), Iron chloride hexahydrate (FeCl_3_·6H_2_O, 99%, Macklin, Shanghai, China).

### 2.2. Methods

#### 2.2.1. Preparation of the Thermal-Sensitive Hydrogel

An amount of 10 g of PNIPAM is dissolved in 16 mL toluene. The solution is heated to 60 °C with stirring at 400 rpm in a magnetic stirrer water bath until completely dissolved. Next, 40 mL of n-hexane is added to the solution and stirred for another 20 min at 60 °C. Toluene and n-hexane are added twice more. The mixture is filtered to remove undissolved impurities, and the filtrate is left to stand in a -20 °C freezer for 12 h. After filtration, white flocculent crystals are obtained, yielding 7 g of PNIPAM crystals after freeze-drying, with a 70% yield. Next, 0.015 g of BIS and 0.015 g of TPO are sequentially added to 10 mL of deionized water and stirred for 10 min. XLG is added in batches to the solution under stirring within 10–30 s, followed by continued stirring for 60 min until the solution forms a gel. PNIPAM and DMAPMA are added to the mixed solution and stirred for 2 h under nitrogen protection. The solution is then transferred to a centrifuge to remove air bubbles from the gel, with a centrifuge speed set at 6000 rpm for 5 min, yielding a thermosensitive deformable hydrogel printing solution suitable for extrusion-based 3D printing. The ink formula contains PNIPAM, DMAPMA, BIS, TPO, and XLG. The modeling software is Solidworks 2022. After establishing the model, the model is saved as a file with the suffix stl format for later use. The model of the 3D printer is a FOODBOT-D1 extrusion printer (SHNINOVE, Hangzhou, China), and the slicing software is Repetier-Host Windows 2.3.2. Import the model file in stl format into the RepetierHost software to adjust the appropriate printing position and size, then slice the model—this process is to transform the 3D model into a G-code motion path that can be recognized by a 3D printer. Before printing, the printing speed was adjusted to 20 mm/s, the diameter of the print head was about 0.8 mm, and the printing fluid extrusion speed was 10 mm/s. Finally, the printed and formed samples were subjected to ultraviolet curing.

#### 2.2.2. Preparation of Magnetic Hydrogel

Amounts of 4.0 g (15 mmol) FeCl_3_·6H_2_O and 1.5 g (7.5 mmol) FeCl_2_·4H_2_O were added to 100 mL deionized water, and the solid particles were stirred at a rate of 500 rpm for 20 min to completely dissolve into an iron salt ion aqueous solution with a Fe^2+^:Fe^3+^ molar ratio of 1:2. NaOH solutions with concentrations of 1 M, 2 M, 3 M, 4 M, and 5 M were prepared, respectively. The printed thermosensitive gel was soaked in deionized water for 48 h to remove unreacted impurities. Then, it was transferred to 80 °C hot water to shrink and eliminate expansion. After the swelling, the gel was fully immersed in the iron salt ion aqueous solution to fully swell, allowing Fe^2+^ and Fe^3+^ ions to diffuse into the gel polymer network through the action of water molecules; the process lasted 36 h. Subsequently, the gel was transferred to the solution of NaOH, causing the solution and the gel to quickly turn black. After 24 h of sufficient absorption and reaction, magnetic Fe_2_O_3_ particles were formed in situ within the gel network. Since the Fe^2+^ and Fe^3+^ ions had already migrated into the hydrogel network before contacting the NaOH solution, the hydroxide ions followed along with water molecules during the migration process to generate magnetic Fe_2_O_3_ particles in situ within the gel network. Thus, a magnetic gel with magnetic particles inside the gel network is formed, and this method is in situ precipitation [25].

### 2.3. Characterizations

The mechanical properties of hydrogels were tested by the electronic universal testing machine Model 5965 of the American Instron Company (Norwood, MA, USA). At room temperature, the dumbbell-shaped samples were tested with pneumatic fixtures, 5 groups of each sample were tested, and the tensile rate parameter used in the test was 2 mm/min. X-ray Photoelectron Spectroscopy (XPS, K-alpha type, Thermo Fisher Scientific company in the Hamptons, NH, USA) was used to analyze the element composition of modified hydrogel surface and the surface of the micromorphological change process, explore the surface chemical properties of the obtained surface, and fit the high-resolution spectra of the characteristic elements. The back-bottom vacuum of the vacuum chamber used in the test was 1 × 10^−8^ bar, and the X-ray source was Al Kα (12 kV; 6 mA). Scanning Electron Microscope (SEM) tests are characterized by scanning electron microscopy (SU8000, Hitachi, Tokyo, Japan). Since the test object is not conductive, the sample needs to be sprayed with gold in advance. The Transmission Electron Microscope (TEM) analysis was performed on a Talos F200X G2 (Shanghai, China) transmission electron microscope operating at an accelerating voltage of 200 kV. For SEM and TEM analyses, the magnetic hydrogels were first dehydrated by vacuum freeze drying (2KBTXL, VirTis, Los Angeles, CA, USA). The magnetic hysteresis loops of the Fe_2_O_3_/PNIPAM magnetic hydrogels were analyzed using the Vibrating Sample Magnetometer (LAKE SHORE, Carson, CA, USA) with the maximum applied magnetic field of 31 kOe. The X-Ray Diffraction (XRD) test uses the X-ray diffractometer produced by BRUKER (Billerica, MA, USA). Thermogravimetric analysis (TG) of the magnetically responsive hydrogel was performed with thermal gravity (RT-1500 °C, Selb, Germany) under a nitrogen atmosphere at a heating rate of 5 °C min^−1^. The Fourier Transform Infrared Spectrometer (FTIR) was recorded on a Fourier Infrared Spectrometer (IS50, THERMO, Shanghai, China). Rheological testing of the hydrogel was carried out using a rotary rheometer (Mars 40, HAAKE, Vreden, Germany).

## 3. Results and Discussion

### 3.1. Morphology Characterization of Magnetic Gel

**Figure 1:**
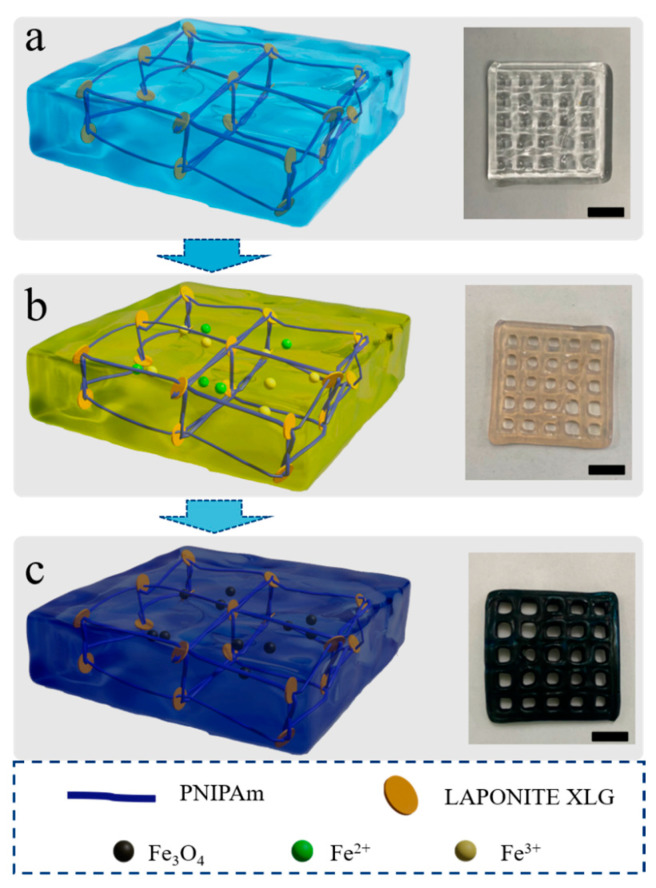
3D printing process of magnetic gel. (**a**) 3D-printed temperature-sensitive PNIPAM hydrogel. (**b**) PNIPAM hydrogel soaked in iron ion solution. (**c**) Magnetic hydrogel formed after soaking in NaOH solution.

The process of forming magnetic hydrogel is illustrated in Figure 1. As depicted in Figure 1a, the method begins with the printing of PNIPAM hydrogel samples with a certain microarray structure using the PNIPAM hydrogel printing technique with XLG, as described in the methods of this paper, and the sample appears as a colorless and transparent gel. In Figure 1b, the printed PNIPAM gel is immersed in a deionized water solution containing iron ions (Fe^2+^, Fe^3+^), resulting in the sample turning pale yellow after immersion. As depicted in Figure 1c, after immersion, the sample is treated with a NaOH solution to obtain a black magnetic hydrogel.

PNIPAM is a widely utilized negative thermosensitive polymer [26]. It exhibits good responsiveness to temperature changes, biocompatibility, and adjustable mechanical strength [27]. If the temperature is increased above the Lower Critical Solution Temperature (LCST), water molecules leave the polymer chain and form a globule structure. The polymer changes from a loose coil structure to a tight colloidal structure, so it has the shrinkage of the PNIPAM hydrogel. As a result, the PNIPAM hydrogel is dehydrated, and a definite volume phase transition is observed [28]. Figure S1 depicts the swelling test of temperature-sensitive and magnetic hydrogels. During the heating process, the quality of the hydrogel continues to decrease, indicating that the hydrogel continues to shrink with the increase in temperature. The final shrinkage rate of the temperature-sensitive hydrogel is 57.3%, and the shrinkage rate of the magnetic hydrogel is 43.4%. Due to the existence of magnetic particles, the shrinkage rate of magnetic hydrogel is obviously not as good as that of temperature-sensitive hydrogel. As the temperature increases, the temperature-sensitive hydrogel layer has a better deformation effect. According to the different shrinkage rates of each layer, the directional deformation of the bilayer hydrogel can be achieved in a magnetic field. Therefore, to achieve the obvious deformation process, we designed a bilayer hydrogel structure.

### 3.2. 3D Printing of Magnetic Gel

The optical photo of the printing wire under a high-power microscope at different printing rates is shown in Figure S2, while Figure S3 presents the discrete diagram at various rates. It can be observed that when the printing speed of the 3D printer is set to 10 mm/s, it exhibits low dispersion but results in thicker printing lines, thereby increasing both the thickness and width of the printed layer. This may lead to a deterioration in the accuracy of 3D printing molding, as it prevents proper exposure and curing of edges, causing overlap between adjacent printing layers. Conversely, when employing a printing speed of 30 mm/s, thinner print thickness and narrower width are achieved. However, this also leads to significant stretching and thinning of the printed liquid along with increased dispersion, resulting in compromised printing accuracy. By setting a moderate value for the printing speed (20 mm/s), both print thickness and width remain within acceptable ranges while maintaining reasonable dispersion levels. Consequently, this paper adopts a printing speed of 20 mm/s to ensure smoothness during operation, as well as to achieve the desired precision.

In the extruded 3D printing hydrogel technology in this work, the role of the photoinitiator is to initiate the solidification and formation of the 3D-printed hydrogel. In this paper, five common photoinitiators are compared, namely, 819, 184D, 1173, TPO, and 2959. The five photoinitiators were each dissolved in deionized water to create solutions with a concentration of 3 wt.%. The absorbance of these solutions was measured at wavelengths ranging from 200 to 500 nm, all at a temperature of 20 °C. It can be seen from Figure S4 that the absorption peak widths of the five initiators are from large to small: 819 > TPO > 184D > 1173 > 2959. Among them, the absorbance of photoinitiators 184D, 1173, and 2959 decreases significantly at around 400 nm, while photoinitiators 819 and TPO still have strong absorption above 400 nm, so TPO and 819 are alternative photoinitiators. The solubility of the photoinitiator in water was then tested at 20 °C. The results are presented in Figure S5, and the solubility of the photoinitiator TPO is slightly better than 819. Additionally, the absorption spectra of five photoinitiators at different concentrations were tested, with concentration gradients of 0.1, 0.5, 0.8, 1.0, 3.0, and 5.0 wt.%, the results are shown in Figure S6. According to the Beer–Lambert law, the absorbance of the solution should increase linearly with the increase in concentration. It can be seen from Figure S6e that with the increase of concentration, the absorbance of the photoinitiator TPO shows a gradually increasing trend and appears more stable, and the absorbance curves of other photoinitiators are unstable and perform worse than the photoinitiator TPO. We also compared the absorbance of five photoinitiators at different concentrations at the wavelength of 365 nm, as shown in Figure S6f. It can also prove that the photoinitiator TPO is more stable. As the concentration of TPO increases, its absorbance changes little. Based on the comprehensive evaluation of the absorption performance, solubility, and stability with photoinitiator concentration of five photoinitiators, TPO was ultimately chosen as the photoinitiator in this study.

**Figure 2:**
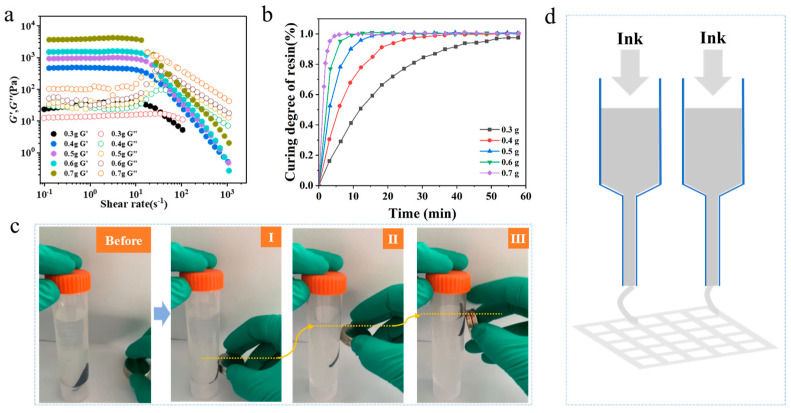
(**a**) Rheological performance curve of LAPONITE XLG solution. (**b**) Relationship curve between curing time and curing degree of different XLG contents. (**c**) Magnetic response of the magnetic hydrogel. The yellow line represents the movement of the magnetic hydrogel. (**d**) Extrusion 3D printing schematic.

For the sake of assessing the printability of XLG solutions with varying concentrations, rheological tests were conducted on solutions containing different amounts of XLG nanoparticles. Specifically, 0.3 g, 0.4 g, 0.5 g, 0.6 g, and 0.7 g of XLG nanoparticles were individually added to 10 mL of deionized water; frequency sweep tests using a rotational rheometer were performed to measure the storage modulus (G′) and loss modulus (G″) of solutions with varying XLG concentrations. It can be seen from Figure 2a that under different concentrations, the same weak strain overshoot LAOS III type behavior is exhibited. Particularly, at low shear rates, both the loss modulus and the storage modulus remain stable under different concentrations. As the shear rate increases, they show different decreasing trends, indicating shear thinning. Meanwhile, it can be observed from Figure 2a that at low shear rates, G′ gradually increases with the increase in concentration. Conversely, G″ gradually decreases with the increase in concentration, which indicates that the higher the content of XLG, the more the printing liquid tends to be solid. This large difference between G′ and G″ implies a higher setting ability, which is beneficial for the shaping of the printing liquid after extrusion from the needle.

Combined with the tilt angle test in Figure S7, when the content of XLG is less than 5 wt.%, the angle between the liquid surface and the container wall decreases after being tilted and left to stand. This is because when the content of XLG is low, the printing liquid precursor retains some degree of self-flowability and poor shape retention, preventing it from setting quickly. It cannot support itself during printing, leading to an inability to achieve precise 3D printing. When the XLG content is 5 wt.% or higher, the angle between the liquid surface and the container wall remains at 90 degrees even after being tilted and left to stand for 10 min, indicating significant improvement in shape retention and stability. This shows that when the content of XLG is 5 wt.% or higher, rapid setting and self-support can be achieved. However, when the addition of XLG is excessive, the printing liquid becomes more viscous, and its fluidity deteriorates, making it impossible to achieve precise printing and shaping. Therefore, in the subsequent experiments of this study, the addition of XLG is set at 5 wt.%.

The relationship curve between gelation time and pre-gelation degree of PNIPAM hydrogels with different XLG contents is shown in Figure 2b, the required gelation time significantly decreases with an increase in XLG content. The magnetic response of the magnetic hydrogel is shown in Figure 2c, where the magnetic hydrogel rod is lifted by being attracted by an NdFeB alloy permanent magnet to overcome its gravity. The extrusion 3D printing principle is shown in Figure 2d. The process of extruding the 3D printing hydrogel can be seen more clearly and intuitively in Figure S8.

### 3.3. Structural Characterization of Magnetic Gel

**Figure 3:**
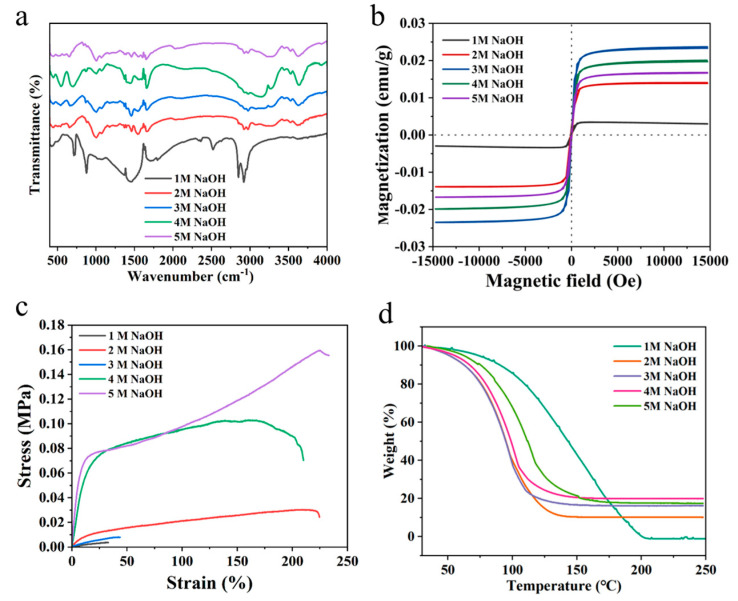
(**a**) The FTIR spectra of magnetic hydrogels obtained by treatment with NaOH aqueous solutions of varying concentrations. (**b**) Magnetic hysteresis loops of magnetic hydrogels treated with different NaOH concentrations. (**c**) Stress–stretch curves of magnetic hydrogels. (**d**) T_g_ test of magnetic hydrogels treated with different NaOH concentrations.

The Fourier Transform Infrared (FTIR) spectroscopy of magnetic hydrogels obtained by treatment with NaOH aqueous solutions of varying concentrations is shown in Figure 3a. All five magnetic hydrogels have clear absorption peaks 1500~1600 cm^−1^ of the amide functional group in the IR spectrum, and there is no significant hydrolysis observed in the magnetic hydrogels obtained by this method. The freeze-dried magnetic hydrogels were then ground into powder and tested for hysteresis loops. As can be seen from Figure 3b, the magnetic properties of magnetic hydrogels prepared at different NaOH concentrations were investigated. When the NaOH concentration is 1 M, the residual magnetization and coercivity decrease to 0.22 emu/g and 1.95 Oe, respectively, exhibiting clear superparamagnetic behavior at room temperature. The saturation magnetization of the thermosensitive magnetic hydrogels increases with increasing NaOH concentration, reaching a maximum value of 4.98 emu/g at 3 M NaOH solution.

Subsequently, with further increase in NaOH concentration, the residual magnetization decreases, possibly due to particle aggregation resulting from higher NaOH concentration leading to a decrease in paramagnetic behavior. However, this decrease is not significant. Standard dumbbell-shaped samples soaked in 1 M, 2 M, 3 M, 4 M, and 5 M NaOH solutions were subjected to tensile fracture tests, as shown in Figure 3c; the tensile strengths were measured as 0.07 N, 0.22 N, 0.24 N, 0.57 N, and 1.26 N, and the elongation at break was recorded as 4.58 mm, 8.43 mm, 28.58 mm, 30.94 mm, and 53.89 mm, respectively. With the increase in NaOH concentration, both the tensile strength and elongation at break significantly increased. The higher concentration of NaOH leads to the in situ precipitation of more magnetic iron oxide particles in the polymer network, and the increase of magnetic particles during the tensile process can disperse the stress of the polymer network to a certain extent, giving the gel itself higher mechanical strength. Therefore, comprehensively considering the magnetic properties and mechanical properties of the hydrogels, this study adopts 5 M NaOH for the preparation of magnetic hydrogels.

As shown in Figure 3d, magnetic hydrogels prepared from 1 M NaOH solution, the thermal gravimetric (T_g_) completely decomposes at around 200 °C with almost zero residue. This is probably because at lower NaOH concentrations, there were very few magnetic Fe_2_O_3_ particles produced, and after heating, the PNIPAM polymer completely decomposed. The decomposition temperatures for 2 M, 3 M, 4 M, and 5 M NaOH solution-treated magnetic hydrogels are 136 °C, 140 °C, 142 °C, and 156 °C, with residual weights of 10.16%, 16.26%, 19.89%, and 17.54%, respectively. After increasing the NaOH concentration, the content of magnetic Fe_2_O_3_ particles produced by in situ precipitation significantly increases, with decomposition temperatures around 140 °C. The T_g_ results show a weight change plateau after heating to 140 °C, where the remaining mass is the magnetic particles within the gel that cannot thermally decompose. The residual weight generally increases with the increase in NaOH concentration, indicating that within the 1–5 M range, higher NaOH concentrations help increase the iron oxide content. It is worth noting that the residual weight of 5 M in the thermal gravimetric results, 17.54%, is slightly lower than the residual weight of 19.89% for 4 M. This may be because the in situ precipitated magnetic Fe_2_O_3_ in the PNIPAM polymer network can reach a maximum content, and excessively high NaOH concentrations may cause the agglomeration of the magnetic particles already in the polymer network into larger particles, leading to inadequate adhesion to the gel network and subsequent detachment.

**Figure 4:**
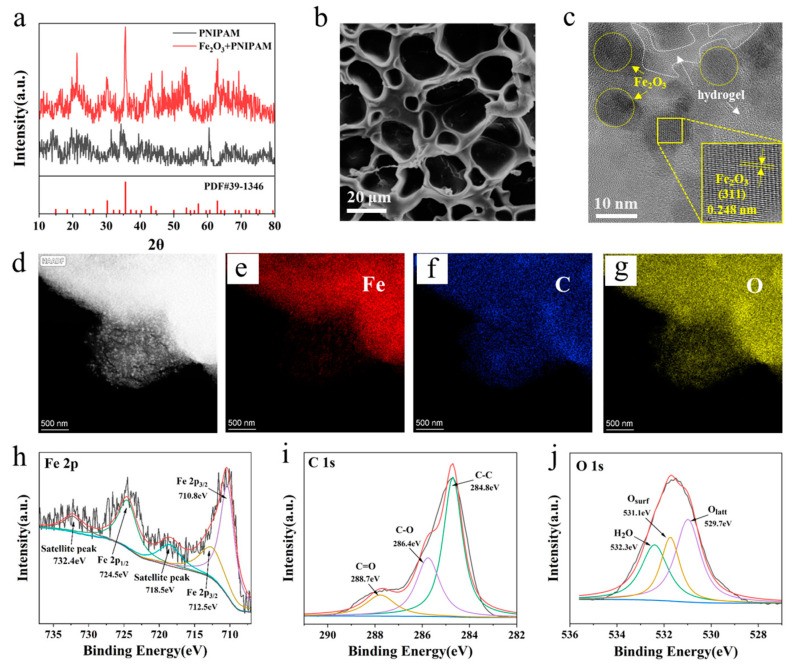
(**a**) XRD test of PNIPAM temperature-sensitive hydrogels and Fe_2_O_3_ + PNIPAM magnetic hydrogels. (**b**) Magnetic hydrogel SEM structure diagram. (**c**,**d**) TEM images of magnetic hydrogels. (**e**) Fe, (**f**) C, and (**g**) O TEM mapping of magnetic hydrogels. (**h**) Fe 2p, (**i**) C 1s, and (**j**) O 1s XPS analysis of magnetic hydrogels.

Figure 4a exhibited XRD patterns of PNIPAM temperature-sensitive hydrogel and Fe_2_O_3_ + PNIPAM magnetic hydrogel at the range of 20–80° (2θ). All the characteristic diffraction peaks of Fe_2_O_3_ + PNIPAM magnetic hydrogels were well agreed with the standard pattern of magnetic Fe_2_O_3_ particles (JCPDS card No. 39-1346), demonstrating the existence of Fe_2_O_3_ in the hydrogel system [29]. To further investigate the structural and surface elements of magnetic hydrogels, SEM and TEM images of magnetic hydrogels were recorded. Figure 4b is the SEM image showing the 3D polymer network of the magnetic hydrogel. From Figure 4c, the lattice fringes of 0.248 nm were in accordance with the spacing (311) plane of magnetic Fe_2_O_3_ [29], and the magnetic Fe_2_O_3_ particles are highlighted with yellow circles and yellow arrows, while the hydrogel is indicated by a white border and white arrows. Therefore, it could be concluded that the magnetic Fe_2_O_3_ particles were successfully fabricated according to the results. Moreover, the chemical compositions of magnetic hydrogels were analyzed by element mapping, and exhibited in Figure 4d–g, the results indicated that Fe, C, and O coexisted within the sample. The uniformly distributed black Fe_2_O_3_ particles in the magnetic hydrogel can be more clearly seen in Figure S9. To further study the chemical states and compositions of the magnetic hydrogels, an X-ray photoelectron spectroscopy (XPS) spectrum was carried out. The spectra in Figure 4h show the Fe 2p_3/2_ peak at 710.8 eV and 712.5 eV of Fe(III), the Fe 2p_1/2_ peak at 724.5 eV for γ-Fe_2_O_3_, the peak at 718.5 eV belongs to the satellite peak of Fe 2p_1/2_, and the peak at 732.4 eV belongs to the satellite peak of Fe 2p_3/2_ [30]. As shown in Figure 4i, there are three peaks located at 284.8, 286.4, and 288.7 eV, which correspond to C−C, C−O, and C=O, respectively [31]. Figure 4j shows the O 1 s XPS peaks, the O 1 s peaks were fitted into three peaks at 529.7, 531.1, and 532.3 eV, which were defined as the lattice oxygen (O_latt_), surface adsorbed oxygen (O_surf_), and adsorbed molecular oxygen (H_2_O), respectively [32].

### 3.4. 4D Deformation of Magnetic Gel

**Figure 5:**
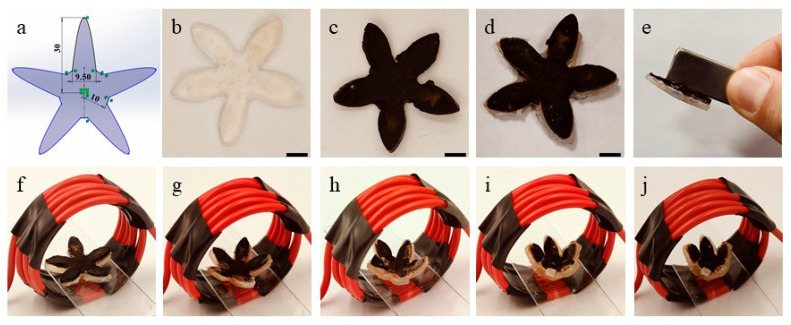
4D-printed bilayer hydrogel. (**a**) Solidworks design 3D model of five-pale flower. (**b**) 3D-printed five-pale flower hydrogel. (**c**) Magnetic five-pale flower hydrogen. (**d**) Bilayer hydrogel. (**e**) Magnetic test of the bilayer hydrogel. (**f**–**j**) 4D deformation of the bilayer hydrogel. Red circles are 5 mm copper coils (diameter 5 mm, 6 turns, 65 A). (Scale bar is 10 mm).

To demonstrate the actual 4D deformation process of the magnetic hydrogels, a five-petal flower-shaped structure was designed using Solidworks, as shown in Figure 5a, and the length from the center to the tip of the petal is 30 mm with a thickness of 2 mm. As shown in Figure 5b, a single-layer flower-shaped hydrogel was printed using extrusion 3D printing technique with PNIPAM and XLG. As shown in Figure 5c, the printed magnetic hydrogel was prepared with the method mentioned in 2.2.2. Another single-layer hydrogel was printed and bonded to the black magnetic hydrogel to create a bilayer hydrogel (Figure 5d), and its magnetic properties were tested using a magnet (Figure 5e). Subsequently, the bilayer magnetic hydrogel was placed on a microscope slide, inserted into an electromagnetic field (65 A, wire diameter 5 mm with 6 strands), and the deformation process was observed and recorded (Figure 5f–j). As shown in Figure 5f–j, the entire deformation process was finished within 300 s, and a photo interval of approximately 75 s.

## 4. Conclusions

In summary, we have developed a 3D printing technology for in situ generated magnetic hydrogels. Thermal-responsive PNIPAM hydrogels were prepared using 3D printing with the in situ-formed Fe_2_O_3_ magnetic particles in the PNIPAM network to generate the magnetic hydrogel, and the magnetic Fe_2_O_3_ particles are evenly distributed in the magnetic hydrogel. The fabricated bilayer hydrogel, comprising a temperature-sensitive layer and a magnetic-responsive layer, can undergo a shape transformation within a magnetic field. The 4D-printed bilayer five-pale flowers finished the deformation process within 300 s.

## Data Availability

The original contributions presented in this study are included in the article/Supplementary Material. Further inquiries can be directed to the corresponding authors.

## Supplementary Materials

The following supporting information can be downloaded at: https://www.mdpi.com/article/10.3390/nano15020134/s1.
