# Supplementary material for: 4D-Printed Magnetic Responsive Bilayer Hydrogel
Source: Nanomaterials (Basel). 2025 Jan 17;15(2):134. doi: 10.3390/nano15020134 (PMC11767551; doi:10.3390/nano15020134)
Supplement: Supplementary file 1 [file nanomaterials-15-00134-s001.zip › nanomaterials-3374972-supplementary.docx]

Supplementary Information

4D printed magnetic responsive bilayer hydrogel

Yangyang Li^1,#^, Yuanyi Li^1,#^, Jiawei Cao^1,2^, Peng Luo^2^, Jianpeng Liu^2^, Lina Ma^3^, Guo-Lin Gao^1,*^, Zaixing Jiang^1,*^

^1^ MIIT Key Laboratory of Critical Materials Technology for New Energy Conversion and Storage, School of Chemistry and Chemical Engineering, Harbin Institute of Technology, Harbin 150001, P. R. China.

^2^ JiangHuai Advance Technology Center, Hefei 230009, P. R. China.

^3^ College of Chemistry and Chemical Engineering, Qingdao University, Qingdao 266071, P. R. China.

^#^ Y. Li and Y. Li contributed equally to this work.

***** Correspondence: gaoguol@hit.edu.cn (G.-L. G.), jiangzaixing@hit.edu.cn. (Z. J.)


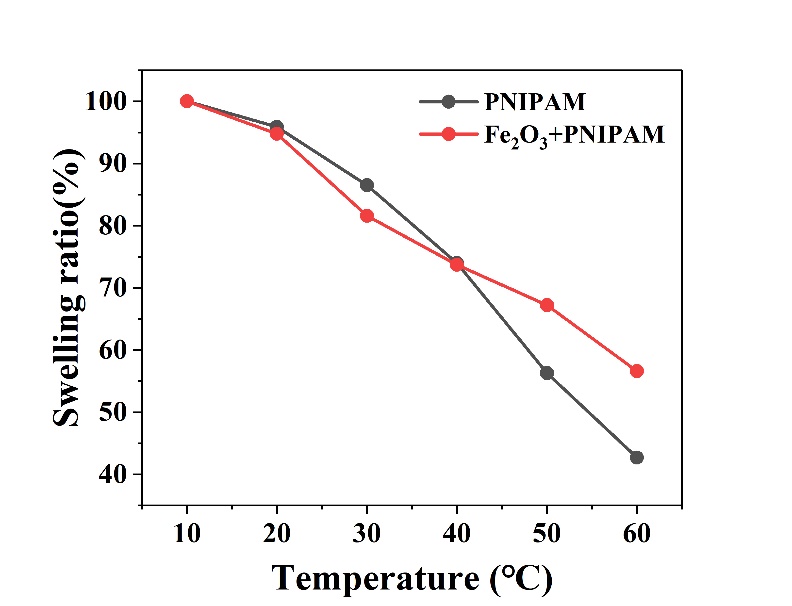


Figure S1. The swelling ratio curve of temperature-sensitive hydrogels and magnetic hydrogels


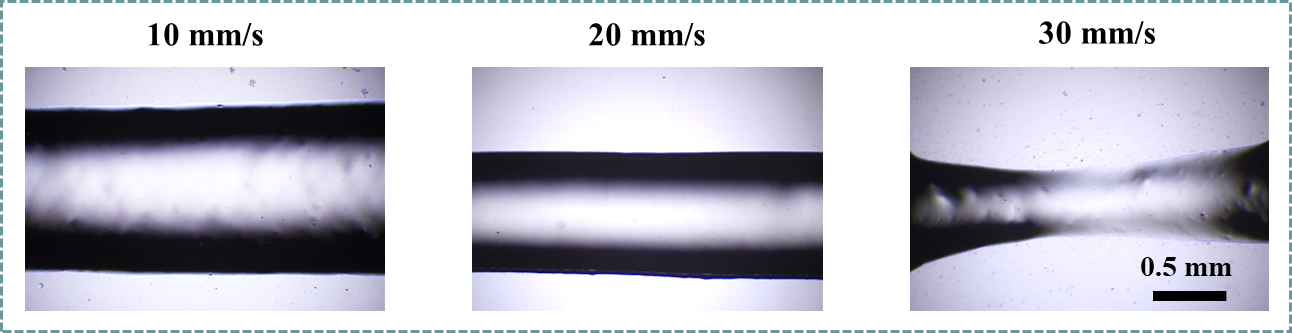


Figure S2. The optical photo of the printing wire under a high-power microscope at different printing rates


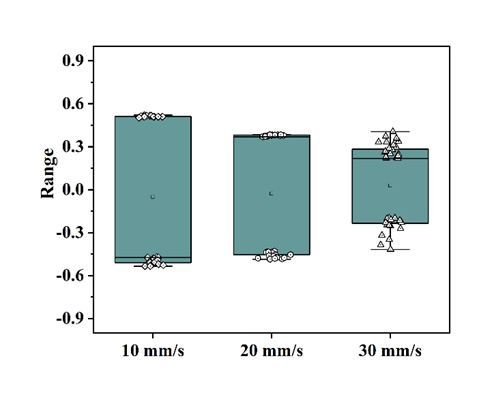


Figure S3. Discrete plots of different print speeds


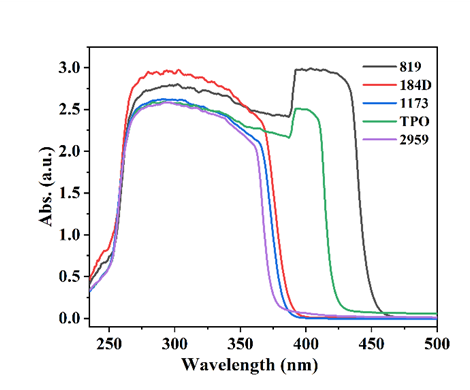


Figure S4. Full absorption spectrum of five photo-initiators


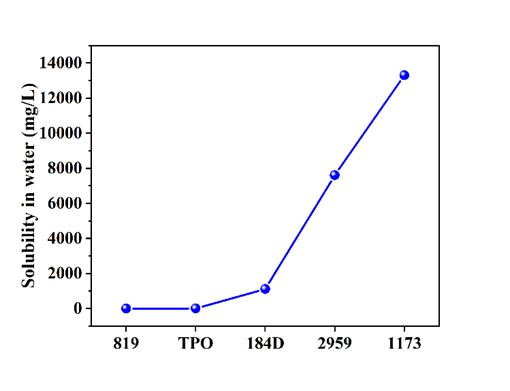


Figure S5. Comparison curves of solubility of five photo-initiators


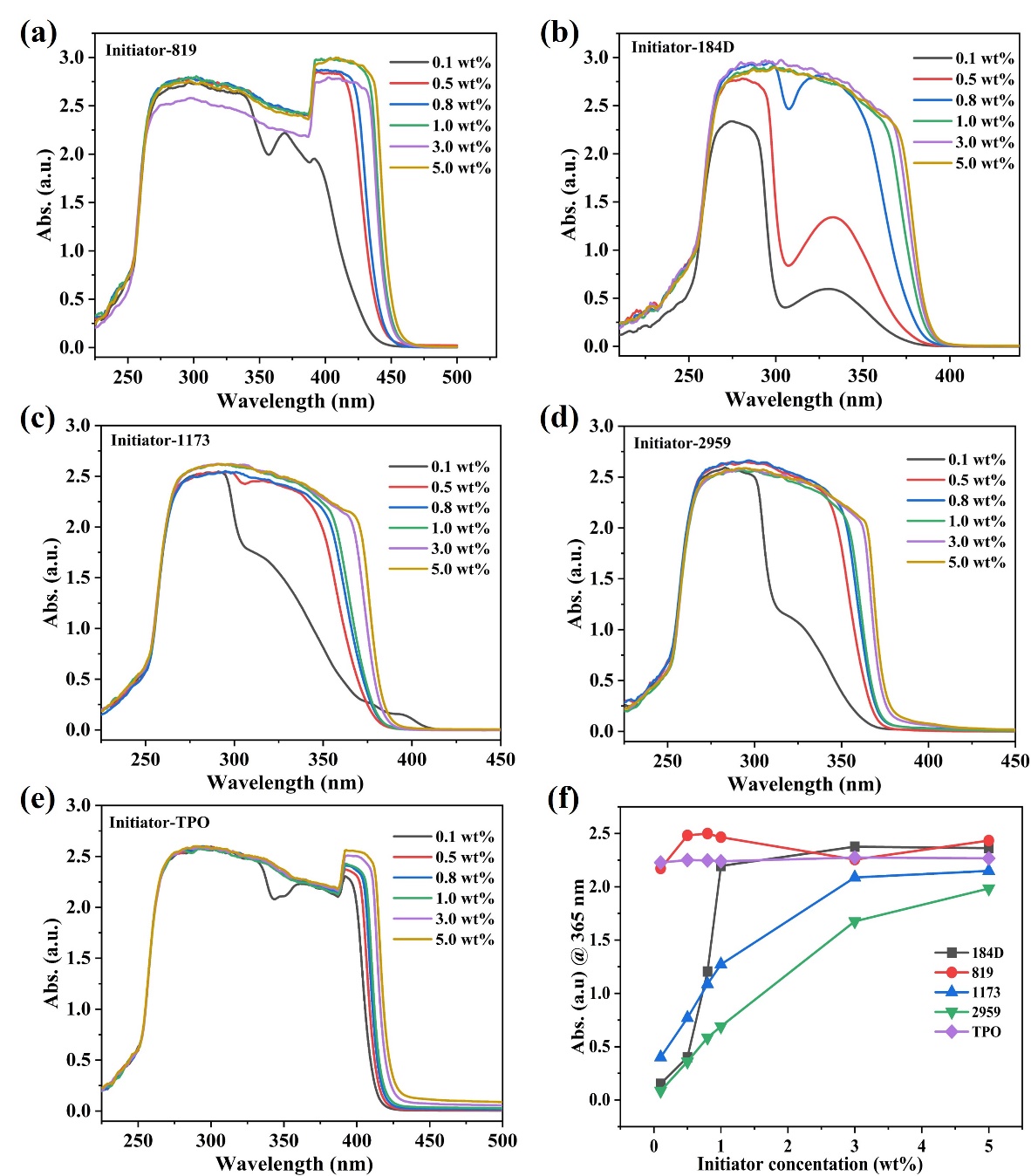


Figure S6. UV-Vis absorption spectra of different concentrations of five photo-initiators. (a) Initiator 819. (b) Initiator 184D. (c) Initiator 1173. (d) Initiator 2959. (e) Initiator TPO. (f) Absorbance at 365 nm wavelength of five photoinitiators with different concentrations.


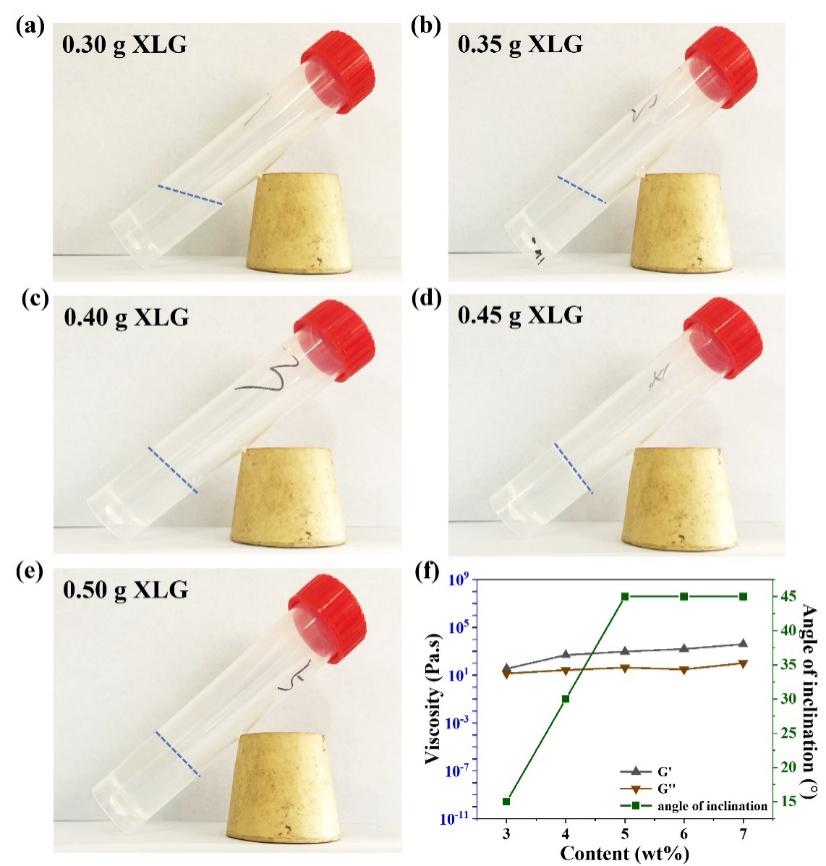


Figure S7. Experiment on static tilt angles of printing fluid precursors with different XLG contents in 10 mL solution. (a) 0.30 g XLG. (b) 0.35 g XLG. (c) 0.4 g XLG. (d) 0.45 g XLG. (e) 0.5 g XLG. (f) Tilt angle curves of different XLG contents.


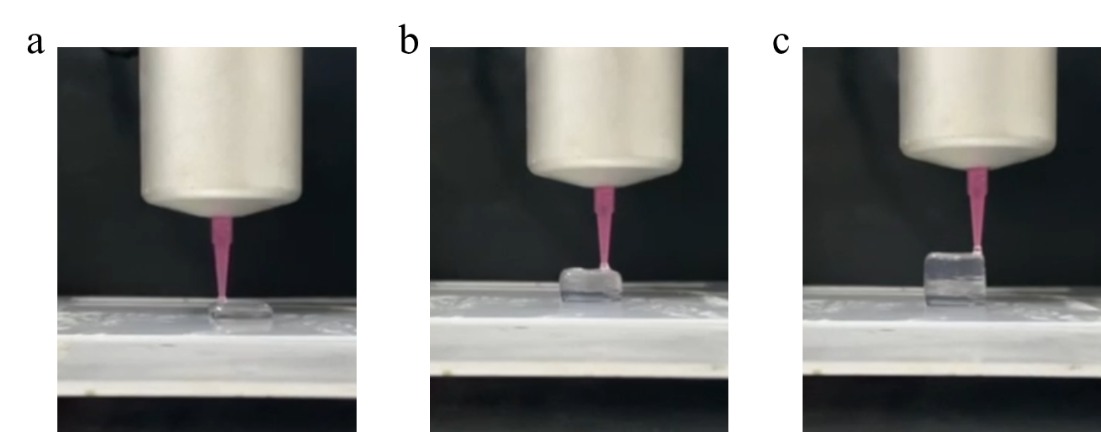


Figure S8. The process of extruding 3D printing hydrogel. (a) 3D printing process begins. (b) Intermediate process of 3D printing. (c) 3D printing process complete.


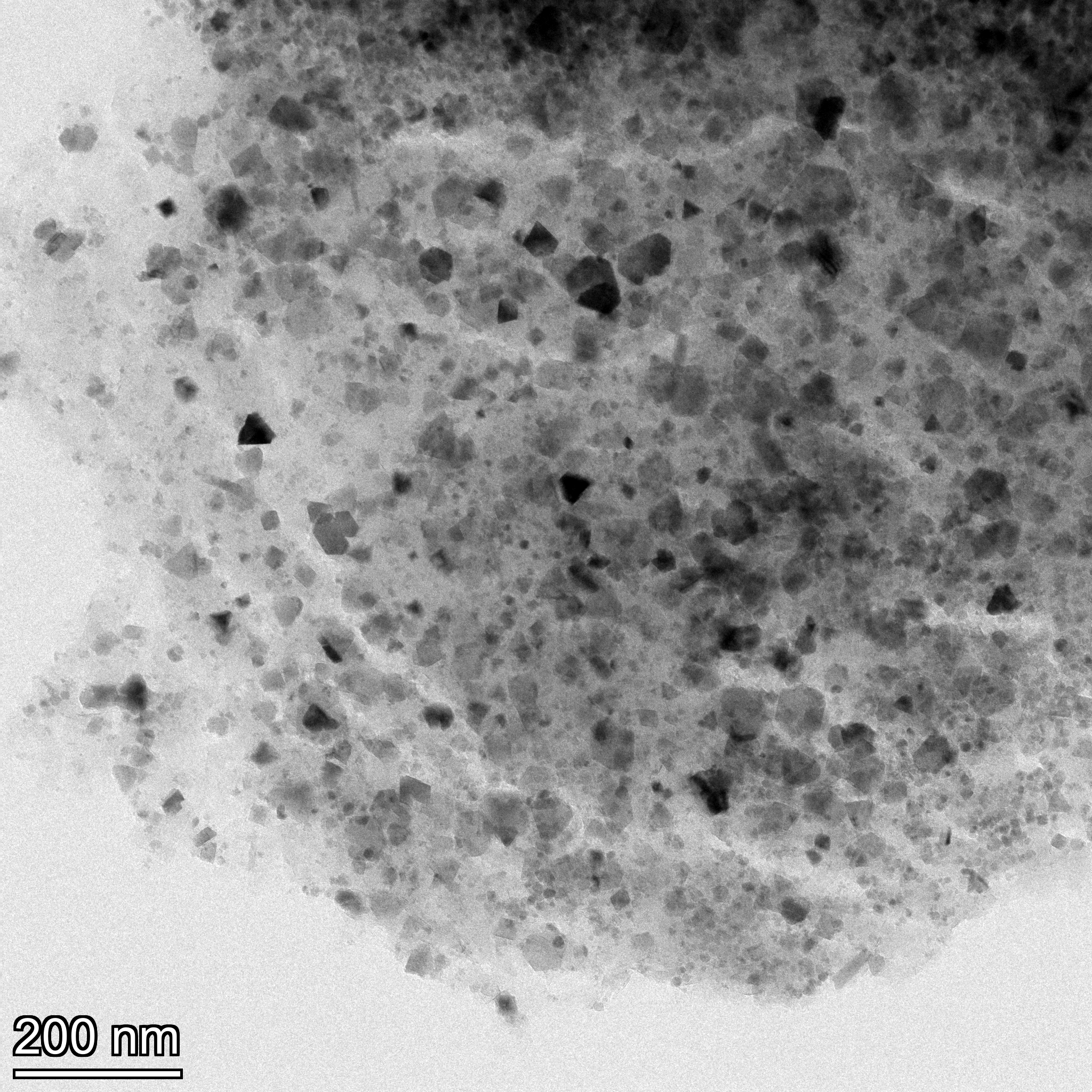


Figure S9. TEM image of magnetic hydrogel
